# Introducing a Neuroscience-Based Assessment Instrument: Development and Psychometric Study of the Neural Networks Symptomatology Inventory

**DOI:** 10.1177/00332941241226685

**Published:** 2024-01-17

**Authors:** Bruno Faustino, Isabel Fonseca

**Affiliations:** Lusófona University, HEI‐Lab: Digital Human‐Environment Interaction Labs, Portugal; 37809 Faculdade de Psicologia da Universidade de Lisboa, Lisboa, Portugal; 37809 Faculdade de Psicologia da Universidade de Lisboa, Lisboa, Portugal

**Keywords:** Neural network symptomatology inventory, Neuroscience, Psychometrics, Exploratory factor analysis, Confirmatory factor analysis

## Abstract

**Background:** Neuroscience research methods contribute to the understanding of the underlying neural impairments associated with psychopathology. Previous research suggested that impairments in Default Mode Network, Fronto-Parietal Executive Network, Amygdaloid-Hippocampal Memory Network, and Attentional Salience Network are present in different psychopathological symptoms. However, a self-report measure based on this evidence is lacking. **Aims:** Therefore, the present study describes the development and preliminary psychometric study of the Neural Network Symptomatology Inventory (NNSI). *Method*: Two different samples were recruited (sample 1: *N* = 214, Mage = 21.0, SD = 7.10; sample 2: *N* = 194, Mage = 21.5, SD = 8.41) and responded to self-report instruments in a cross-sectional design. Standard methodologies to scale development and psychometric study were applied: Item development, Exploratory (EFA), Confirmatory Factor Analysis (CFA), and Pearson correlations. **Results:** EFA and CFA suggested a 4-factor model with adequate goodness-of-fit indices (χ2(449) = 808,9841, TLI = .89, CFI = .92, RMSEA = .048 (.042–.053). All NNSI subscales correlated positively with psychopathological domains and correlated negatively with psychological well-being. **Conclusions:** This preliminary study suggests that NNSI may be a valid instrument to assess symptomatology associated with complex neural network impairments. Nevertheless, further research is required to deepen and improve NNSI psychometric characteristics.

## Introduction

The integration between psychotherapy and neuroscience has been suggested as a pathway to understanding multifactorial interactions between psychological and neurobiological domains associated with psychological disorders (Cacioppo et al., 2015; Cozolino, 2017; Grawe, 2007; Kandel, 1998). Nevertheless, understanding associations between different levels of analysis may be a complex task. Recently, efforts were made to propose several neuroscience-informed principles to enhance psychotherapy responsiveness, along with a theoretical description of how several neurobiological systems may be underlying emotional needs, interpersonal motivations, and life themes (Faustino, 2022a, 2022b). Beyond theoretical conceptualizations, the integration between psychotherapy and neuroscience may also be achieved by the development and empirical testing of self-report instruments based on the neural basis of personality and symptomatology. Thus, several neuroscience-based assessment instruments are available in the literature, and some examples may be given.

The Affective Neuroscience Personality Scale (ANPS, Davis et al., 2003) is a self-report instrument developed to assess behavioral manifestations and experiences associated with six Neurobiological Affective Systems (ANS), namely, ANGER, SADNESS FEAR, PLAY, SEEKING and CARE SYSTEMS - LUST SYSTEM was not added to the ANPS to avoid response biasis (Davis & Panksepp, 2004). The ANS measured by the ANPS showed theoretically congruent correlations with the Temperament Evaluation of Memphis, Pisa, Paris, and San Diego Autoquestionnaire (TEMPS-A; Akiskal et al., 2005), Cloninger’s Temperament, Character Inventory (TCI; Cloninger et al., 1993) and Five-Factor Model (FFM, McCrae & Costa, 2008). Nevertheless, some limitations have been found, especially in the factorial structure (Barret et al., 2013). The ANPS has 112 items and was considered too long, which motivated the development of a short form, the Brief Affective Neuroscience Personality Scales (BANPS), which presented adequate psychometric properties in the original form (Barret et al., 2013) and in other cultural adaptations (Faustino et al., 2023). These instruments are focused on the assessment of neuroscience and/or neurobiological constructs related to personality. Nevertheless, other assessment instruments are focused on the assessment of neuropsychological symptoms related to brain disorders.

The Dysexecutive Questionnaire (DEX; Burgess et al., 1996) is a self-report instrument with 20 items focused on the assessment of neuropsychological symptoms related to the dysfunctionality of the frontal lobe processes following an acquired brain injury (ABI). It has two versions, one for the self (DEX-S) and the other for one informant (DEX-I). The first Exploratory Factor Analysis (EFA) of the DEX-S showed a three-factorial model differentiating symptoms related to emotion, behavior, and cognition. However, subsequent studies showed that the dimensionality of the DEX-S proved to be complex without a coherent factorial structure (Loschiavo-Alvares et al., 2013). Following a psychometric study done by Simblett and Bateman (2011), a new revised version was developed based on an Rach-Analysis showing a coherent four-factor structure detailed as metacognition/social cognition, executive cognition, behavioral-emotional self-regulation and activation (DEX-R, Simblett et al., 2017). Comparative validity and reliability of both versions (DEX-I and DEX-S) were tested in the four scales with adequate psychometric properties.

The Behavior Rating Inventory of Executive Function–Self-Report version (BRIEF-SR, Gioia et al., 2015) is a self-report instrument to assess executive functioning in adolescents. It has 80 items divided into eight nonoverlapping clinical scales that represent different executive functions (inhibition, shifting, emotional control, monitoring, working memory, planning, organization, and task completion scales). Warren et al., (2021), through EFA and CFA, detailed a three-factorial structure for the BRIEF-SR in affective disorders. Other instruments were also developed with an underlying rationale based on brain functioning processes, such as the Everyday Memory Questionnaire—revised (EMQ-R, Royle & Lincoln, 2008), Multifactorial Memory Questionnaire (MMQ, Troyer & Rich, 2002), Brock Adaptive Functioning Questionnaire (BAFQ, Dywan & Segalowitz, 1996), Behavioral Assessment of the Dysexecutive Syndrome (BADS; Wilson et al., 1998), and Working Memory Questionnaire (WMQ, Vallat-Azouvi et al., 2012). All respective studies suggested adequate measurement properties, which means that self-report instruments may also be an asset in neuropsychological assessment to assess neurocognitive symptomatology without the cost of time and money of a formal battery. Nevertheless, there is an ongoing debate in the literature regarding this issue but is beyond the scope of the present paper (Wennerhold & Friese, 2020).

The described instruments have proven to be useful in assessing specific traits and symptoms based on the structural approach to brain functioning. However, recent models based on neuroscience methods, such as Functional Magnetic Imaging (fMRI), showed that brain functioning may be better explained with connectionist neural models (Guest & Love, 2017;Guest & Martin, 2021). Complex neural networks (CNN) are systems of neural pathways from different brain structures connected by nodes, which support complex psychological functions. The tripartite model of psychopathology (Menon, 2011) emphasizes the notion that several CNN may be related to mental health. Malfunction in specific CNN means that impairments in neural communication between distinct parts of the brain may explain several symptoms related to core psychopathological manifestations. According to Menon (2011), three functional neural networks are essential to mental health and psychopathology: Fronto-Parietal Executive Network (FPEN), Attentional Salience Network (ASN), and Default-Mode Network (DMN). However, recently, Faustino (2022c), proposed that the Amygdaloid-Hippocampal Memory Network (AHMN) may also be a CNN implicated in psychopathology and theorized that malfunctions in these four CNN may support a new neuropsychopathological syndrome underlying psychopathology. Previous research suggested that individuals with diagnostics of depression, anxiety, and schizophrenia spectrum disorders showed behavioral manifestations that may be associated with these four CNN malfunctions. In Table 1, a revision of specific CNN functions, structures, impairments, and supporting studies is described. However, this conceptualization remained theoretical. In this sense, this study aims to start the empirical validation of the neuropsychopathological syndrome through the development of the Neural Networks Symptomatology Inventory (NNSI). Specifically, the present paper describes: (a) the development of a scale based on a coherent neuroscience-based model, (b) which clusters several neuropsychopathological symptoms congruently, (c) in a parsimoniously brief self-report measure, and (d) tested empirically with psychometric analysis.

**Table 1.** Brief description of neural networks and related domains, structures, functions, symptoms, disorders, and supporting studies (adapted from Faustino, 2022c).

Neural Network	Main Neuroanatomical Structures	Neurocognitive Functions	Psychological Symptoms	Psychopathological Disorder	Supporting Studies
Frontal-parietal executive network	Dorsolateral prefrontal cortex (DPC)	Executive functions (inhibition, cognitive flexibility updating, abstract reasoning) and self-regulation	Apathy; loss of focus, planning, and abstract abilities, difficulties in goal-directed behavior	Depression spectrum disorders; bipolar disorder; schizophrenia; substance use disorders	Faustino et al. (2021); Snyder (2013); Snyder et al. (2015); Robinson & Ferrier (2006); Yang et al. (2017)
	Superior and inferior parietal lobes (SIPL)	Working memory actualization, visuospatial navigation, sensorial integration	Difficulties in visual-spatial problem solving, mental imagery and somatosensory integration deficits		
Attentional salience network	Anterior insula (AI)	Detection of emotion/sensory-based internal and external stimuli	Difficulties in performance and task monitoring, salience confusion	Anxiety disorder, posttraumatic stress disorder; alcohol use disorders	Galandra et al. (2018); Geng et al. (2016); Mor & Winquist (2002); Shi et al. (2019)
	Dorsal anterior cingulate cortex (DACC)	Stimuli response discrimination and conflict prioritization and resolution	Difficulties in insight and emotion-based complex decisions; risk-based decisions		
Amygdaloid hippocampal memory network	Amygdala (A)	Emotional fear-based conditioned learning, sensorimotor responses	Hyperactivation of amygdala responses,excessive stress and fear	Anxiety, depression spectrum disorders; bipolar disorder; posttraumatic stress disorder; schizophrenia	Airaksinen et al. (2005); Behnken et al. (2010); Forbes et al. (2009); Martino et al. (2011); Taconnat et al. (2010)
	Hippocampal cortex (HC)	Memory formation, encoding, and retrieval	Memory perseveration and stagnation,stereotyped memory		
Default mode network	Medial prefrontal cortex (MPC)	Self-related decision making, autobiographical memories, and future aims	Difficulties in self-awareness, reflection, referencing, and self-other discrimination, impaired sense of self	Depression spectrum disorders; anxiety disorders; posttraumatic stress disorder; schizophrenia	Coutinho et al. (2016); Daniels et al. (2011); Doucet et al. (2020); Lee et al., 2018; Yan et al. (2019); Zhou et al. (2020)
	Posterior cingulate cortex (PCC) and precuneus (P)	Bottom-up attentional processing with perception and memory stimuli; self and self-other thinking, memories, and concepts, visual and sensorimotor processing	Social and emotional disinhibition, impaired social cognition and sensorimotor integration		
	Angular gyrus (AG)	Integration of attention, perception, and spatial cognition	Difficulties in passive perception, mental spatial navigation, and prospection		

## Study 1 - Development of Neural Network Symptomatology Inventory

The purpose of Study 1 was to develop the Neural Network Symptomatology Inventory (NNSI) and to explore basic psychometric properties. As stated before, the need for a psychometrically valid instrument based on a coherent neuroscience approach to psychopathological symptoms is a long-standing issue in neuroscience and psychopathology literature.

## Methods

### Sample

Two different samples were used for the development of the NNSI. Sample one consists of 214 participants where 38 were male (17.8%) and 176 were female (82.2%), with an age amplitude from 18 to 49 years (Mean = 21.0, SD = 7.10). Sample two consists of 194 participants where 30 were male (15.5%) and 164 were female (84.5%), with an age amplitude from 18 to 65 years (Mean = 21.5, SD = 8.41) – see Table 2

**Table 2.** Characterization of two samples.

		Sample 1	Sample 2
*N*		214	194
Age			
	*M*	21.0	21.5
	*SD*	7.10	8.41
	Minimum	18	18
	Maximum	49	65
Gender (freq - %)			
	Male	38 (17.8%)	30 (15.5%)
	Female	176 (82.2%)	164 (84.5%)
Nationality (freq - %)			
	Portuguese	202 (94.4%)	180 (92.8%)
	Brazilian	6 (2.85)	9 (4.6%)
	Mozambican	2 (.9%)	2 (1.0%
	Italian	-	1 (.5%)
	Colombian	-	1 (.5%)
	French	1 (.5%)	1 (.5%)
	Romenian	1 (.5%)	-
	Ukrain	1 (.5%)	-
	Dominican rep	1 (.5%)	-
Education (freq - %)			
	12 grade	171 (79%)	164 (93.8%)
	Bachelor	35 (16.4%)	20 (10.3%)
	Master/PhD	8 (3.8%)	10 (5.1%)
Relationship status (freq - %)			
	Single	201 (93.9%)	182 (93.8%)
	Married	11 (5.1%)	9 (4.6%)
	Fact union	1 (.5%)	1 (.5%)
	Divorced	1 (.5%)	2 (1.0%)
Psychotherapy (freq - %)			
	Yes	63 (29.4%)	54 (27.8%)
	No	151 (70.6%)	140 (72.2%)

#### Procedures

The first sample was recruited in the academic year of 2021/2022, and the second sample in the academic year of 2022/2023 from an undergraduate psychology course from the Faculty of Psychology of the University of Lisbon. Individuals had one week to complete all questionnaires hosted on the Qualtrics platform. Individuals received credit for participation. Research aims and purposes were explained at the beginning of the study, and it was clearly stated that all responses were mandatory. It was also stated in the beginning that individuals could withdraw at a given moment with prejudice to receive a credit bonification. To explore data validity, four procedures were used: (1) time completion to avoid random/fast responses (below 15 minutes were excluded and higher than 1 hour); (2) all 1 or all 5 responses were eliminated; (3) duplicate IPs were removed and (4) only full participations were considered valid (Aust et al., 2013). In the first sample, 21 participations were considered invalid based on the above criteria. Initially, a small sociodemographic questionnaire was presented with closed questions (yes or no) about self-report diagnosis (e.g., “do you have a diagnosed neurocognitive disorder?”). Inclusion criteria were the following: being over 18 years old, speaking Portuguese as a native language (or speaking for more than 5 years), and not having a neurodevelopmental and/or neurodegenerative disorder. The present research was approved by the ethics committee and deontology of the Faculty of Psychology of the University of Lisbon. This study was not preregistered. All study procedures were aligned with Helsinki Convention.

#### Statistical Analysis

First, descriptive statistics were used to explore and detail sociodemographic variables. Second, several items corresponding to symptomatology associated with the four neural network dysfunctions described previously were generated (DMN, FPEN, ASN, and AHMN). Third, an Exploratory Factor Analysis (EFA) was performed to explore the factorial structure of the NNSI. Fourth, basic psychometrics were studied through internal consistency, if-item deleted, and inter-item correlation. The normality of the variables was checked through skewness and kurtosis indexes. Internal consistency was evaluated using Cronbach’s alpha, α ≥ 60 was considered acceptable (George & Mallery, 2003). The average inter-item correlation was considered acceptable within values between .15 and .50 (Paulsen & BrckaLorenz, 2017). All analyses were performed in the SPSS 25 version. 

## Results

### Item Development

As stated before, the development process of the NNSI followed Boateng et al., (2018) suggested guidelines.

Step 1 Identification of the domain(s) and item generation

The first step was to identify the specific symptoms that are associated with the four neural network dysfunctions, which are the domains for the NNSI. Table 1 describes the most prevalent symptomatology associated with DMN, FPEN, ASN, and AHMN. After the domain identification, a pool of items was generated accordingly: (1) based on a literature review of measures that can be associated with symptoms related to CNN dysfunctions; (2) measures with adequate psychometric properties; (3) content analysis, and (4) expert consensus. The analysis of similar instruments was used to produce an item pool without coping exactly the same items. Thus, they were used as an inspiration to formulate new items for the NNSI.

A literature review of similar measures was carried out to explore the best items to fit the domains of the NNSI. To develop the DMN subscale, the Dissociate Experiences Scale (DES, Bernstein & Putnam, 1986) was consulted because DMN dysfunctions are systematically related to difficulties in self-experiences and integration, self-referential cognition, and differentiation, which is very similar to dissociative symptomatology. To develop the FPEN subscale, the Diexecutive Questionnaire Revised (DEX-R, Simblett et al., 2017) was explored because FPEN impairments underly executive dysfunctions which are closely associated with dyexecutive syndrome. Core executive function impairments associated with diexecutive syndrome tend to be inhibition, inflexibility, stickiness, and difficulties in decision-making and problem-solving (Diamond, 2013). To develop the ASN subscale, the Everyday Life Attention Scale (ELAS, Groen et al., 2019) was consulted. ELAS is a self-report instrument focused on the assessment of attentional difficulties of different situations in daily life. Individuals are asked to report their level of attention in situations like reading a book, having a conversation, and seeing a movie. To develop the AHMN subscale, the Memory Assessment Clinics Self-Rating Scale was consulted (MAC-S, Crook & Larrabee, 1990). MAC-S is a self-report instrument aiming to access abilities and the occurrence of memory difficulties daily. Finally, the Brief Symptoms Inventory-53 (BSI-53, Derogatis, 1993), was also considered to explore item format, wording, and syntax. In this sense, item wordings were based on the affirmative notion of the presence of the symptoms by the responder. Therefore, based on the literature review, 100 items were generated to match the 4 neural networks (25 per domain).

Step 2 Content validity

To ensure content validity, an expert analysis was conducted by the following criteria: (1) acceptance of the behavioral content; (2) ambiguity; (3) relevancy to symptomatic measurement; (4) experts’ agreement; and (5) reliably observed (Guion, 1977). The scale with 100 items was assessed by two experts (Ph.D. professors with degrees in clinical psychology and neuroscience with more than five years). The scale was also filled out by 10 independent individuals with knowledge of the measure, to assess vocabulary clarity and comprehensiveness. Fifty-six items were dropped due to the lack of consensus by the experts in item wording, ambiguity of the corresponding construct, the complexity of the item, and observable content. Eleven items were re-worded for vocabulary clarity. Finally, the NNSI with 54 items was ready to be presented to a larger sample.

### Exploratory Factor Analysis of the NNSI

A sample with 214 participants was used to explore the factorial structure of the NNSI. The extraction of the factors was based on eigenvalues higher than 1. The Varimax rotation procedure was chosen, rather than the Oblimin procedure rotation, because it is expected that all subscales represent different CNN symptoms that share common real neural connections (Watkins, 2018). Maximum likelihood estimation was used in EFA. Thirteen factors were extracted, explaining 61.10% of the total variance. Items below .40 were removed, and the factorial solution converged in 19 interactions. The Kaiser-Meyer-Olkin measure presented was .876, revealing a good correlation between the variables (Pestana & Gageiro, 2008). Bartlett’s sphericity index was statistically significant [χ^2^ (7796), gl = 1431, *p*. <.001], revealing an adequate correlational pattern within the items (Pestana & Gageiro, 2008).

Factor 1 explained 28,15% of the variance and clustered 9 items (51, 54, 50, 49, 52, 48, 53, 45, and 46), describing difficulties in the experience of the self, such as item 50 – “*My behaviors do not match what I think and feel*” (self-incoherence) or item 51 – *“I confused about what I am as a person”*, (identify fragmentation) which are associated with DMN dysfunctions. Factor 2 explained 6.70% of the variance and clustered 7 items (1, 2, 7, 3, 12, 6, and 13), describing difficulties in executive functions, such as item 1 – “*I´m not able to change what I am thinking even if I need to*” (cognitive inflexibility) or item 3 – “*I can’t go an idea even when it does not make sense to me*” (lack of updating), which tend to be associated with FPEN dysfunctions. Factor 3 explained 5.67% of the variance and clustered 5 items (37, 36,39, 38, and 16), describing difficulties in memory, such as item 16 – *“I have difficulties in keeping “things” in memory”* (memory codification impairment), or as item 36 – “*I have difficulties in recalling significant events of my personal history*” (episodic memory impairment), which are associated with AHMN dysfunctions. Factor 4 explained 4.36% of the variance and clustered 3 items (26, 27, and 31), describing difficulties in attentional processes, such as Item 26 – *“I’m incapable of concentrating on a task for many hours”* or 27 – “*I have difficulties in concentrating in the actions that I must do”* (both expresses focused attention impairment), which is associated with ASN dysfunctions. Factor 5 explained 3.15% of the variance and clustered 3 items (29, 28, and 30), describing difficulties in attentional processes, such as Item 29 – *“I’m unable to do different tasks at the same time”* or 30 – “*I focus my attention in one task at a time”* (both expresses difficulties in divided attention), which is associated with ASN dysfunctions. Factor 6 explained 2.48% of the variance and clustered 3 items (4, 8, and 9), describing difficulties in executive functions of perspective-taking/decentration, such as Item 4 – *“I have difficulties in thinking on different alternatives for the same situation”* or item 9 – *“Usually, I only have one way of seeing things”,* which tend to be associated with FPEN dysfunctions. Factor 7 explained 2.29% of the variance and clustered 3 items (10, 11, and 14), describing difficulties in executive functions of impulse control/regulation, such as item 10 – *“I have difficulties in controlling my impulses”* or item 11 – *“When I am angry, I can’t control myself*” which tend to be associated with FPEN dysfunctions. Factor 8 explained 1.84% of the variance and clustered 2 items (18 – *“I take too much time in doing plans”* and item 19 – *“I have many difficulties in taking decisions”*), describing difficulties in executive functions of planning and decision-making which tend to be associated with FPEN dysfunctions. Factor 9 explained 1.72% of the variance and clustered 3 items (32, 33, and 35), describing difficulties in past meaning-making, such as item 33 – *“I have difficulties in differentiating the meanings of my life*” or item 35 – *“I cannot understand the meaning of some of my past experiences*”, which can be associated with DMN dysfunctions. Factor 10 explained 1.52% of the variance and clustered 3 items (20, 43, and 44), describing difficulties in self-other differentiation and emotionality, such as item – 20 *“My emotions “get in my way” when I have to take an important decision”,* or item 43 – *“I tend to be excessively involved with people who are important to me”*, which may be associated with DMN dysfunctions. Factor 11 explained 1.16% of the variance and clustered 3 items (23, 40, and 41), representing difficulties in self-other differentiation and future planning, such as item 23 – *“I am a person who barely thinks in the future”* or item 40 – *“I have difficulties in differentiating my ideas, opinions, and sensations from others”,* which can be associated with DMN dysfunctions, Finally, factors 12 and 13 were not interpreted, as only one item loaded in both factors.

When a significant proportion of the explained variance drops from the first factor to the others, other factorial options may be explored (Watkins, 2018). In this sense, another EFA was performed forcing the extraction of 4 factors and clustering the 4 neural networks detailed previously. Thus, forcing theoretically coherent factors in EFA is a standard practice in scale development in psychology (Costello & Osborne, 2005). Four factors were extracted explaining 43.82% of the total variance. Items below .40 were removed and the factorial solution converged in 19 interactions. The Kaiser-Meyer-Olkin and Bartlett’s sphericity were the same as the first EFA (KMO = .876; BS = χ^2^ (7796), gl = 1431, *p*. <.001). Factor 1 explained 27.87% of the variance and clustered 10 items (45, 46, 47, 48, 49, 50, 51, 52, 53, and 54), matching a coherent factor representing difficulties associated with DMN dysfunctions. Factor 2 explained 6,45% of the variance and clustered 15 items (1, 2, 3, 4, 6, 7, 8, 9, 10, 11, 12, 13, 14, 20, and 22), matching a coherent factor representing executive impairments associated FPEN dysfunctions. Factor 3 explained 5.34% of the variance and clustered 8 items (16, 32, 33, 35, 36, 37, 38, and 39) matching a coherent factor representing memory symptoms associated with AHMN dysfunctions. Finally, factor 4 explained 4.15% of the variance and clustered 7 items (19, 25, 26, 27, 28, 29, and 30), matching a coherent factor representing attentional symptoms associated with ASN dysfunctions – see Table 3.

**Table 3.** Exploratory factor analysis of the Neural Networks Symptomatology Inventory (NNSI).

	1	2	3	4
51. I am confused as to what I am	0.85			
54. Sometimes I have trouble understanding myself as a person	0.84			
49. I have trouble defining myself as a person	0.83			
50. Sometimes I don’t feel like myself	0.82			
48. Sometimes I feel like a stranger in my own skin	0.77			
52. I have trouble understanding what I need as a person	0.77			
53. I have trouble identifying with some of my thoughts, feelings, and behaviors	0.75			
45. I feel confused about what I think, feel, and do	0.62			
46. My behaviors are not in line with what I really think and feel	0.56			
47. I have ideas and sensations that are not part of the way I see the reality around me	0.48			
40. I have difficulty distinguishing my ideas, feelings, and opinions from those of others				
41. I have difficulty feeling like an individual person and different from others				
2. I have difficulty in modifying the contents of my thinking		0.78		
7. Even when necessary I can’t change what I think		0.75		
6. I find it very difficult to stop thinking about certain things		0.74		
1. I am not able to change what I am thinking even if I need to		0.73		
12. I find it very difficult to inhibit unwanted thoughts		0.67		
3. I am not able to let go of an idea, even when it doesn’t make sense for me to have that same idea		0.67		
13. I can’t inhibit unpleasant emotions and feelings		0.62		
10. I have difficulty controlling my impulses		0.52		
11. When I am angry I can’t control myself		0.47		
9. I usually only have one way of looking at things		0.45		
22. I have trouble solving my problems		0.44		
14. I don’t hold back verbally even when I know I’m going to hurt myself		0.44		
4. I find it very difficult to think of several alternatives for the same situation		0.44		
20. My emotions get in the way when I have to make an important decision		0.44		
8. I can’t think of several alternative explanations for situations that happen to me when I’m not expecting it		0.42		
5. I consider myself a stubborn person				
15. I am incapable of letting go of a preconceived idea				
43. I get excessively involved with the people who are important to me				
42. My thoughts, feelings and needs may be the same as other people’s				
36. I don’t remember most of the important episodes in my life			0.79	
37. I have difficulty remembering situations from my past			0.79	
39. I dimly remember the important events I experienced with my family			0.76	
38. I am not able to adequately relate a past encounter/event with a friend			0.73	
16. I have trouble keeping “things” in memory			0.55	
32. I have trouble remembering some of the meanings of past situations	0.43		0.48	
33. I have difficulty differentiating the meanings of my experiences	0.40		0.44	
35. I can’t understand the significance of some situations that I have experienced in the past			0.41	
34. I have difficulty interpreting the meanings of popular proverbs				
23. I am a person who thinks very little about the future				
28. I can’t do several tasks at the same time				0.88
29. I am not able to do several different things simultaneously				0.85
26. I am unable to concentrate on a task for many hours				0.58
27. I have trouble concentrating on the actions I have to do				0.57
30. I focus my attention on one task at a time				0.51
25. I have difficulty following a conversation when several people are talking at the same time				0.45
19. I find it very difficult to make decisions	0.41			0.42
18. It takes me a long time to make plans				
31. I am unable to control my attention and end up seeing and hearing things I don’t want to				
24. I easily lose my attention in a conversation				
17. I am a disaster at doing head calculations				
21. I am a person with little commitment to my goals				
44. I am so close to my family that I sometimes have trouble thinking for my head				

### Internal Consistently and Inter-Item Correlation

The four subscales were computed and the internal consistency was explored. The total index of the NNSI with 40 items showed an excellent Cronbach Alfa of .95. DMN subscale with items showed an excellent Cronbach Alfa of .94. FPEN subscale showed an excellent Cronbach Alfa of .91. AHMN subscale showed a very good Cronbach Alfa of .89. ASN subscale showed a good Cronbach Alfa of .84. The average inter-item correlation revealed adequate values in the FPEN (*r* = .39), AHMN (*r* = .50) and in the ASN (*r* = .43). In the DMN subscale, the value average item-correlation value was higher than expected (*r* = .61) based on the criteria described by Paulsen and BrckaLorenz (2017) – see Table 4.

**Table 4.** Correlational analysis for NNSI scales.

	Total Index of NNSI	DMN subscale	FPEN subscale	AHMN Subscale	ASN Subscale
Total index of NNSI	-				
DMN subscale	.66^**^	-			
FPEN subscale	.84^**^	−.12^*^	-		
AHMN subscale	.67^**^	−.14^**^	.27^**^	-	
ASN subscale	.75^**^	−.06^**^	.55^**^	.23^**^	-

Note. * = *p*.<05; ** = *p*.<01.

## Discussion of Study 1

Through revised previous symptomatic neuropsychological scales, it was possible to develop a coherent measure focused on a theoretical neuroscience-based model where dysfunctions on four CNN, namely, FPEN, ASN, DMN, and AHMN, may underlie a hypothesized neuropathological syndrome. This is the first attempt to validate an inventory with this conceptual organization.

First, the initial EFA extraction showed 13 factors that represented different symptoms related to neurocognitive processes. Most factors detailed theoretic congruent symptomatology, such as factor one, which encompassed identity fragmentation and self-incoherence, or factor two, encompassing items resembling cognitive inflexibility. These results support the initial validation of the NNSI by differentiating specific symptomatology based on neurocognitive impairments aligned with theoretical predictions. That is, distinct symptomatology may arise from distinct complex neural impairments, such as identity fragmentation from DMN impairments and cognitive inflexibility from FPEN impairments. Then, a second-order factorial structure was explored to test if the four-factor hypothesized structure would fit the data. A coherent four-factor clustered different symptoms related to the hypothesized CNN. Only items 32, 33, and 19 cross-loaded in two different factors. Items 32 and 33 represent difficulties in differentiation and meaning-making based on past experiences cross-loaded in the AMHN and DMN. This may indicate a shared neural basis between these two CNN in supporting these processes. Item 19 represents difficulties in the decision-making process cross-loaded in ASN and DMN, and the same explanation may be given. Nevertheless, these three items were kept in the respective factor with the higher loading.

Internal consistency was satisfactory. The correlational matrix between the total index of NNSI and subscales was also satisfactory. Mostly, inter-item correlations matched Paulsen and BrckaLorenz (2017) values. However, the inter-item correlation of the DMN subscale was higher than what was expected. One possible explanation may relate to DMN functions, which may share the same variance across other subscales. The DMN is a CNN that supports higher-order processes related to the self, such as self-decision-making, autobiographical memories, and integration of attention (Cozolino, 2017). These processes may also be related to other CNN, for instance, the decision-making processes tend to be associated with executive functions. Nevertheless, the main difference is that in DMN, these processes tend to be related to the self, and in the FPEN, they tend to be related to instrumental actions. The same can be said for autobiographical memories and the integration of attention.

In the second study, the four-factor structure was confirmed through a CFA in a different sample. Based on standard criteria, several items were removed to achieve an adequate model fit to the data, then the internal reliability was also explored and confirmed. Preliminary convergent validity was tested with correlations with the Brief Symptom Inventory-53 (BSI-53, Derogatis, 1993), and preliminary discriminant validity was also obtained by dividing the total sample by an empirical criterion of the BSI-53.

## Study 2

The purpose of Study 2 was to confirm the factorial structure of the NNSI and to explore convergent validity with other measures focused on symptomatology.

## Method

### Instruments

#### Mental Health Index

The Mental Health Index (MHI-5, translated and adapted to Portuguese by Ribeiro, 2001) is an assessment measure focused on mental health and can be used to explore psychological distress and psychological well-being. It is a self-report instrument with 5 items on a Likert scale of five or six points, where higher values suggest a higher presence of the two constructs described previously. See Cronbach’s alphas in Table 5.

**Table 5.** Descriptive statistics for NNSI scales, BSI-53 general index and subscales, and psychological well-being and distress scales.

	*n*	α	*Amp*	*Min*	*Max*	*M*	*SD*	*SK*	*KS*
Total index of NNSI	194	.89	2.76	1.86	4.62	3.39	.56	−.27	−.27
Default mode network (NNSI)	194	.86	2.69	2.03	4.72	3.80	.48	−.56	.30
Frontoparietal executive network (NNSI)	194	.88	3.83	1.17	5.00	3.30	.88	−.17	−.67
Amigdaloyd-hipocampal network (NNSI)	194	.73	3.83	1.17	5.00	2.82	.75	.19	−.70
Attentional salience network (NNSI)	194	.71	3.00	2.00	5.00	4.03	.61	−.63	.41
Psychological distress (MHI-5)	194	.78	5.00	.00	5.00	2.98	.63	.20	1.17
Psychological well-being (MHI-5)	194	.72	5.00	.00	5.50	3.33	.49	−.90	7.22
General symptoms index (BSI-53)	194	.97	3.64	.04	3.68	1.25	.69	.36	−.33
Somatization (BSI-53)	194	.85	4.00	.00	4.00	.89	.78	.92	.61
Obsessive compulsive (BSI-53)	194	.81	3.83	.00	3.83	1.65	.83	.17	−.57
Interpersonal sensitivity (BSI-53)	194	.82	3.75	.00	3.75	1.44	.92	.28	−.59
Depression (BSI-53)	194	.89	4.00	.00	4.00	1.36	.92	.46	−.46
Anxiety (BSI-53)	194	.85	3.83	.00	3.83	1.34	.85	.52	−.45
Hostility (BSI-53)	194	.78	3.60	.00	3.60	.95	.71	1.02	1.05
Phobic anxiety (BSI-53)	194	.84	3.80	.00	3.80	1.01	.92	.90	.16
Paranoid ideation (BSI-53)	194	.73	3.80	.00	3.80	1.36	.74	.27	−.23
Psychoticism (BSI-53)	194	.75	3.60	.00	3.60	1.20	.82	.49	−.33

Note. *α = Cronbach alpha; Amp = Amplitude; Min = Minimum; Max = Maximum; SD = Standard-Deviation; SK = Skewness; KS = Kurtosis.*

#### Brief Symptom Inventory

The Brief Symptom Inventory (BSI-53; Derogatis, 1993), translated and adapted to Portuguese by Canavarro, 1999), is a psychological instrument able to assess nine symptomatic domains (e.g., depression, anxiety, interpersonal sensitivity) associated with psychopathology. It is a self-report measure with 53 items on a 5-point Likert scale, where higher values in the subscales suggest a higher presence of the construct. Cronbach’s alphas in Table 5.

#### Statistical Analysis

First, descriptive statistics were used to explore and detail sociodemographic variables. Second, a Confirmatory Factor Analysis (CFA) with maximum likelihood estimation was conducted to confirm the first structure of the NNSI. Criteria for CFA were the following: Chi-2 (χ2) with a ratio <5 as acceptable; Comparative Fit Index (CFI) and a Tucker–Lewis index (TLI); with a cut-off ≥.90 as acceptable; Root Mean Square Error of Approximation (RMSEA) with a value <.08 was considered acceptable (Meyers et al., 2016). A sample size higher than 150 is acceptable for CFA (Muthén & Muthén, 2002). All analyses were performed in SPSS 25 and AMOS 27 software versions.

## Results

### Confirmatory Factor Analysis

**Figure 1.**
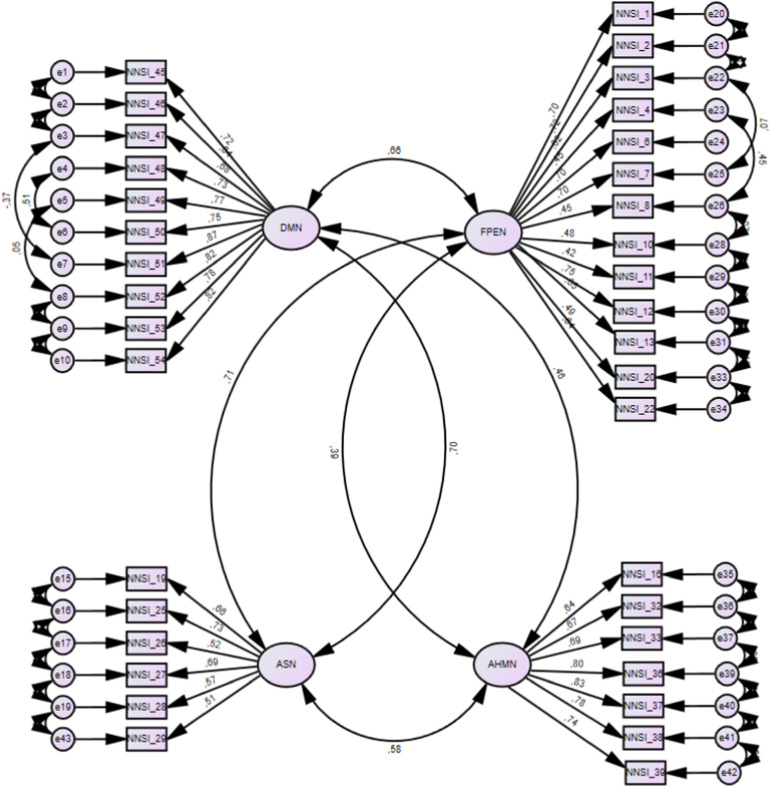
Confirmatory factor analysis of the NNSI.

An initial CFA with maximum likelihood estimation was conducted to confirm the factorial structure of the NNSI. The sample used had194 participants (M = 21.5, SD = 8.41). Results showed the following model fit: χ2 = 1553,477, *df* = 717, TLI = .81, CFI = .82, RMSEA = .078 (.072–.083), which was not an adequate fit to the data (Meyers et al., 2016). To achieve model fit two steps were used: (1) to explore modification indices and (2) to remove factor loadings lower than .40. All errors were allowed to covary across all items and their specific subscales. In this way, it was possible to understand how errors covary depending on the variation in the response to the items. As items belong to the same construct, it is expected that there will be, on the one hand, differences in the response to the item and, on the other, similarities in terms of shared variance. Thus, modification indices increase model fit allowing the estimation of parameters that were not explicit and/or constrained. In the DMN subscale, modification indices suggested covariance errors between items 47 and 51, 48 and 50, and 49 and 52. In the FPEN subscale, modification indices suggested covary errors between items 4 and 8, and between items 26 and 30. Second, items 9 and 14 (FPEN), 30 (ASN), and 35 (AHMN) were removed due to poor factor loading on the construct (<.40). After this procedure, another CFA was conducted with the following indexes: χ2 = 973,889, *df* = 560, TLI = .89, CFI = .92, RMSEA = .062 (.055–.068), which represent adequate fit to the data (Meyers et al., 2016) - see Figure 1. The final form of the NNSI has 36 items: DMN with 10 items, FPEN with 13 items, ASN with 6 items, and AHMN with 7 items – see Figure 1.

### Correlational Analysis

Pearson correlations showed a congruent correlational pattern. The total index of NNSI and all subscales were positively correlated with psychological distress (MHI-5) and all BSI-53 subscales were negatively correlated with psychological well-being (MHI-5). Some examples may be given. The highest strong correlations were found between NNSI total score with Obsessive Compulsive (*r* = .71, *p* < .01), GSI (*r* = .66, *p* < .01), and Psychoticism (*r* = .65, *p* < .01). Also, the DMN subscale have strong correlations with GSI (*r* = .65, *p* < .01), Depression (*r* = .65, *p* < .01) and Obsessive Compulsive (*r* = .64, *p* < .01). The lowest correlation was found between AHMN Subscale and psychological distress (*r* = .16, *p* < .01) – see Table 6.

**Table 6.** Correlational analysis for NNSI scales, BSI-53 general index and subscales, and psychological well-being and distress scales.

	Total Index of NNSI	DMN subscale	FPEN subscale	AHMN Subscale	ASN Subscale
*Mental Health Index-5*					
Psychological distress	.41**	.41**	.38**	.16*	.32**
Psychological well-being	−.44**	−.42**	−.39**	−.24**	−.32**
*Brief Symptomatic Inventory-53*					
General symptoms index (GSI)	.66**	.65**	.51**	.40**	.51**
Somatization	.45**	.46**	.32**	.30**	.32**
Obsessive compulsive	.71**	.64**	.51**	.50**	.64**
Interpersonal sensitivity	.63**	.60**	.49**	.40**	.51**
Depression	.61**	.65**	.45**	.35**	.43**
Anxiety	.52**	.55**	.42**	.24**	.41**
Hostility	.51**	.44**	.50**	.31**	.34**
Phobic anxiety	.45**	.43**	.33**	.26**	.41**
Paranoid ideation	.45**	.46**	.36**	.24**	.34**
Psychoticism	.65**	.62**	.50**	.44**	.50**

Note. * = *p*.<05; ** = *p*.<01.

#### T-Test for Independent Sub-Samples

The cutoff value (1.7<) from BSI-53, which is an empirical criterion of individuals with clinical symptomatology (Canavarro, 1999), was used to divide the full sample into two different subsamples: High Symptoms Sub-sample (HSS) and Low Symptoms Sub-sample (LSS). Significant statistical differences were found between HSS and LSS in the total index of NNSI and all subscales (*p* < .01). Higher mean values were found in the HSS – see Table 7.

**Table 7.** T-test comparisons for NNSI subscales in the total sample (N = 408) were divided into low symptom subsample and high symptom subsample (BSI criteria <1.7).

		*N*	Mean	SD	t	Sig
Total index of NNSI	LSS	292	2.82	74	−12.527	.001
	HSS	116	3.81	.66		
DMN subscale	LSS	292	2.62	1.12	−11.385	.001
	HSS	116	4.02	1.10		
FPEN subscale	LSS	292	2.97	.82	−10.072	.001
	HSS	116	3.87	.78		
AHMN subscale	LSS	292	2.34	1.00	−7.478	.001
	HSS	116	3.21	1.17		
ASN subscale	LSS	292	3.21	1.04	−7.782	.001
	HSS	116	4.09	.99		

Note.* LSS = Low Symptoms Sub-sample; HSS = High Symptoms Sub-sample.*

## Discussion of Study 2

Confirmatory analysis was satisfactory for model adjustment after the application of standard practices to improve model fit. Application of improvement practices in new models in CFA analysis is current practice, especially in the development of new self-report instruments (Byrne, 2010). First, visual inspection using modification indices suggested that some error items would improve model fit if they were allowed to covariate. In the DMN subscale, errors of items 47 and 51, 48 and 50, and 49 and 52 were covariate and, in the FPEN subscale, errors between items 4 and 8 and between items 26 and 30 were also covariate. Second, item removal was considered for items 9 and 14 (FPEN subscale), 30 (ASN subscale), and 35 (AHMN subscale), because they showed factor loadings below .40 (Meyers et al., 2016). After these procedures, a second CFA model was tested showing adequate model fit to the data. However, TLI values tend to be sensible to sample size, and the sample in this study, despite being over 150 (*n* = 194), may have had an impact on this index. In this sense, this analysis should be performed in a larger sample to explore if the TLI index changes accordingly.

Correlational analysis showed that items of the NNSI total index and subscales are correlated with all psychopathological symptoms measured with the BSI-53, which suggests a convergent validity of the NNSI. This preliminary result suggests that the NNSI is effectively measuring neuropathological symptomatology that may be associated with CNN dysfunctions in the FPEN, ASN, DMN, and AHMN. Also, these results may support the notion that neurocognitive processes and CNN dysfunctions may be described as transdiagnostic factors for psychopathology (Faustino & Fonseca, 2023). With effect, the BSI-53 item format was used to develop item content for the NNSI because the BSI-53 is one of the most used inventories with excellent psychometric properties. A likely explanation is the straightforward way in which the subscales are written, they tend to minimize the incorrect interpretations of individuals. Therefore, the same formula was applied to the development of the NNSI.

Further construct validity was obtained by dividing the total sample into two different subsamples by the BSI-53 cut-off point, differentiating an HSS and an LSS. Results showed that in the HSS, all mean values of the NNSI were significantly higher than in the LSS, which suggests that symptomatology associated with CNN tends to be higher in individuals with clinically significant symptoms. In this sense, the NNSI seems to be able to capture the intensity of CNN symptomatology in individuals who are experiencing clinically significant psychopathological symptoms, such as somatization, anxiety, depression and/or interpersonal sensitivity. Specific cause and effect links may be theorized, where CNN malfunctioning may not only prompt neurocognitive difficulties but, they can also facilitate psychopathological symptomatology. For instance, Manarte and colleagues (2021) suggested that obsessive-compulsive symptomatology may be related to inhibitory and flexibility EF deficits, while depressive symptoms may be related to EF deficits along with episodic memory and processing speed (McDermott & Ebmeier, 2009). Also, depression and anxiety symptomatology are related to DMN impairments associated with the sense of *self* and reflexive functioning (Doucet et al., 2020; Lee et al., 2018; Yan et al., 2019).

## General Discussion

This is the first study to report the development of a neuropathological inventory focused on a complex neural network perspective of psychopathology. The need for a psychometric valid instrument based on a coherent neuroscience approach to psychopathological symptoms is a long-standing issue in neuroscience and psychopathology literature, which was addressed in the present study. Some limitations will be described. The study samples had more females than males which, may have biased item responses. Also, the study samples were university students which, limits extrapolations for general and clinical populations. Data gathering was online, and although the sample sizes were considered adequate, their potential benefits could be further enhanced with a larger sample size. Therefore, a definitive conclusion about the NNSI and the latent variables cannot be made because this is the first psychometric study of this instrument. Larger samples should be used to replicate these findings and to test measurement invariance. Thus, subsequent studies should address associations between NNSI and similar instruments for each subscale. For instance, instruments such as the DEX-R and the DES should be tested with the FPEN subscale and the DMN subscale respectively. Also, the validity of the NNSI should be tested with other categorial and dimensional measures of symptomatology and personality dysfunction.

Clinicians and researchers may benefit from the use of the NNSI in their clinical practice and investigations. First, symptomatic explorations and descriptions based on coherent models supported by neuroscience methods (Cacioppo et al., 2010; Cozzolino, 2017; Kandel, 1998) may be an asset because it allies different methods of analysis (clinical observations and fMRI) which is in line with contemporary approaches to psychopathology and psychotherapy (Faustino, 2022a;, Menon, 2011). The convergence of methods of analysis increases the validity of clinical and research instruments (Haynes & Lench, 2003). Second, NNSI may facilitate the case conceptualization based on a brain-based approach within a transdiagnostic perspective (Perez, 2023), bypassing the theoretical limitations of a single school of clinical psychology and psychotherapy, which tend to be biased towards its disorder theory model (Norcross & Wampold, 2019). Neurobiology and neurological systems are regarded as key transdiagnostic factors that explain individual differences in psychopathological manifestations (Krueger & Eaton, 2015). In this sense, adopting a neuroscience-based instrument may help clinicians to better understand patients’ phenomenology beyond theoretical orientations. Third, by understanding the cluster of symptoms that are expected due to specific neural network impairment (e.g., DMN impairments), clinicians may, on one hand, adopt a preventive posture by having a strategy to deal with these symptoms and on the other hand, to use specific tasks that would correspond to psychological functions related to the same neutral network impairment (Faustino, 2022a, 2022c). As an example, if an individual manifests a dysfunctional sense of self and difficulties in setting internal boundaries (DMN malfunctioning), clinicians may suggest that the patient reflects on different aspects of self and others, stimulating psychological differentiation (self-reflexive function is also related to DMN). Fourth, a brain-based approach to symptomatology may also facilitate the communication between clinician and patient to help patients better understand themselves and develop adaptive meaning-making to their difficulties in a non-pathological manner.

## Data Availability

Data not shared because this study belongs to an ongoing research project.
